# Comparison of the efficacy and safety of conventional curettage adenoidectomy with those of other adenoidectomy surgical techniques: a systematic review and network meta-analysis

**DOI:** 10.1186/s40463-023-00634-9

**Published:** 2023-03-04

**Authors:** Moayyad Malas, Awwadh A. Althobaiti, Abdullah Sindi, Afnan F. Bukhari, Faisal Zawawi

**Affiliations:** 1 Department of Otolaryngology-Head and Neck Surgery, Faculty of Medicine, King Abdulaziz University, 21589 Jeddah, Saudi Arabia; 2 Department of Surgery - Section of Otolaryngology-Head and Neck Surgery, King Abdulaziz Medical City, Ministry of National Guard Health Affairs, Jeddah, Saudi Arabia

**Keywords:** Conventional curettage adenoidectomy, Surgical techniques, Systematic review, Network meta-analysis

## Abstract

**Objectives:**

There is a lack of robust evidence in regards to whether the intra and post-operative safety and efficacy of conventional curettage adenoidectomy is better than those of other available surgical techniques. Therefore, this study was conducted as a systematic review and network meta-analysis of published randomized controlled trials (RCTs) with the aim of comparing the safety and efficacy of conventional curettage adenoidectomy with all other available adenoidectomy techniques.

**Materials and methods:**

A systematic search of published articles was performed in 2021 using databases such as PubMed/Medline, EMBASE, EBSCO, and the Cochrane Library. All RCTs that compared conventional curettage adenoidectomy with other surgical techniques and were published in English between 1965 and 2021 were included. The quality of the included RCTs have been assessed using Cochrane Collaboration Risk of Bias Tool.

**Results:**

After screening 1494 articles, 17 were identified for comparing several adenoidectomy techniques and were eligible for quantitative analysis. Of those, 9 RCTs were analyzed for intraoperative blood loss, and 6 articles were included for post-operative bleeding. Furthermore; 14, 10, and 7 studies were included for surgical time, residual adenoid tissue, and postoperative complications respectively. Endoscopic-assisted microdebrider adenoidectomy yielded a statistically significantly greater estimate of intraoperative blood loss compared with conventional curettage adenoidectomy (mean difference [MD], 92.7; 95% confidence interval [CI] 28.3–157.1), suction diathermy (MD, 117.1; 95% CI 37.2–197.1). Suction diathermy had the highest cumulative probability of being the preferred technique because it was estimated to result in the least intraoperative blood loss. Electronic molecular resonance adenoidectomy was estimated to be more likely to result in the shortest surgical time (mean rank, 2.2). Participants in the intervention group were 97% less likely to have residual adenoid tissue than children in the conventional curettage group (odds ratio 0.03; 95% CI 0.01–0.15); therefore, conventional curettage was not considered an appropriate technique for complete removal of adenoid tissue.

**Conclusion:**

There is no single technique that can be considered best for all possible outcomes. Therefore, otolaryngologists should make an appropriate choice after critically reviewing the clinical characteristics of children requiring adenoidectomy. Findings of this systematic review and meta-analysis may guide otolaryngologists when making evidence-based decisions regarding the treatment of enlarged and symptomatic adenoids in children.

**Supplementary Information:**

The online version contains supplementary material available at 10.1186/s40463-023-00634-9.

## Introduction

Adenoidectomy, which is the surgical removal of enlarged adenoids, is a commonly performed procedure among children worldwide [1]. Approximately 250,000 adenoidectomy procedures are performed annually by surgeons in the United States [1]. Sleeping problems, nasal airway obstruction, breathing difficulties, frequent otitis media, and chronic rhinosinusitis [2] caused by hypertrophied and sometimes infected adenoids comprise the various indications for adenoidectomy. Over the past decades, different instruments (a steel nail, surgeon’s fingernail, cutting forceps, adenotomes, and adenoid curettes) have been used to surgically remove adenoids [3]. Conventional curettage to remove adenoids was introduced in 1985; since then, it has remained the most commonly performed procedure worldwide [4]. This procedure uses the nasopharyngeal touch method to gauge the adenoid size and determine how the adenoids are related to adjacent structures, thereby helping surgeons to choose the appropriate curette for scraping the adenoid tissue [2, 5].

Diverse surgical instruments and techniques have been used to remove adenoids, including suction diathermy, a microdebrider, an electronic molecular resonance tool, endoscopy, and lasers [6–9]. Currently, endoscopic-assisted adenoidectomy is used by otolaryngologists under general anesthesia, followed by the use of a microdebrider to scrape the adenoid tissue [10]. Although improvements in surgical outcomes have been observed with the invention of new techniques, a plethora of complications can be anticipated [2, 11]. For instance, intraoperative blood loss and postoperative blood loss remain the most common complications of this procedure [2]. Furthermore, postoperative pain, dissatisfaction of the patients, prolonged surgical time, residual adenoid tissue, and infection are some other complications that are inevitable after adenoidectomy [2, 11]. Because the adenoidectomy performance rate is increasing, surgeons need to determine the most suitable surgical technique for their patients [12].

There has always been a debate in the literature regarding whether to perform conventional curettage or endoscopic-assisted adenoidectomy [13]. Most surgeons prefer conventional curettage because it is cost-effective, easily available to patients, and does not require complex instruments [8]. Patients with a low socioeconomic status are better able to afford the cost of conventional curettage; therefore, it is a commonly used procedure in developing countries [8]. However, because it is a blind technique, it can result in injury to the surrounding structures, and there is a high probability of residual adenoid tissue [14]. Individual research studies have shown that conventional curettage is relatively less precise and has more potential for incomplete removal of the adenoids than endoscopic-assisted adenoidectomy [15]. However, some studies have revealed no difference in the results of the two techniques, and few studies have demonstrated that conventional curettage is quicker and better than endoscopic-assisted adenoidectomy in terms of primary hemorrhage and secondary hemorrhage [16]. Despite the many research studies and randomized controlled trials that have been performed to evaluate the safety and efficacy of the different techniques, there is no clear evidence of which technique is best. A meta-analysis conducted in 2016 revealed that endoscopic-assisted adenoidectomy is superior to conventional curettage in terms of blood loss, surgical time, and complications [17]. However, that meta-analysis had several limitations. First, it was a conventional meta-analysis that included only seven studies [17]. Second, it did not assess the efficacy of the methods in terms of residual adenoid tissue and did not differentiate between primary hemorrhage and secondary hemorrhage [17]. Third, it combined all complications as one outcome rather than providing a quantitative synthesis for different types of complications [17]. Finally, it was limited to endoscopic-assisted adenoidectomy and did not compare other techniques such as suction diathermy, electronic molecular resonance adenoidectomy (EMRA), suction cautery with antistick, and gold laser adenoidectomy with conventional curettage [17]. Therefore, we conducted a systematic review and network meta-analysis of all randomized controlled trials with a relatively larger sample size to assess the safety and efficacy of all other techniques and compared them with those of conventional curettage.

## Materials and methods

We performed a systematic review and network meta-analysis to evaluate, synthesize, and combine existing evidence of the efficacy and safety of conventional curettage. Then, we compared the safety and efficacy of conventional curettage with those of all other surgical techniques used to remove the adenoids, in terms of intraoperative blood loss, surgical time, postoperative blood loss, residual adenoid tissue, and postoperative complications. We used the Preferred Reporting Items for Systematic Reviews (PRISMA) guidelines to as well as statement checklist for reporting systematic review involving network meta-analysis (Additional file 1: Supplemental material 1) to undertake this systematic review and network meta-analysis [18].

### Inclusion and exclusion criteria

The inclusion of a study was considered if it was a research study that evaluated the efficacy of conventional curettage and compared it with all other techniques used to remove adenoids among patients 0 to 18 years of age and was published in English through July 2021 across different regions of the world. Additionally, only studies that were randomized controlled trials were included. Cross-sectional studies, qualitative studies, and any other observational studies were excluded. Studies without full texts were also excluded. All studies that consisted of opinions, included criticisms of previous research studies, and editorials were excluded. Studies of nonconventional adenoidectomy and studies that compared the efficacy, safety, and effectiveness of intravenous hemostatic agents injected during the procedure were also excluded.

### Sources of information and search strategy

A systematic search of published articles was performed in 2021. We searched databases such as PubMed/Medline, EMBASE, EBSCO, and the Cochrane Library. References of the selected articles were also screened according to the eligibility criteria for additional related publications. An independent search was performed by three authors who also scanned the results for potentially appropriate studies and retrieved the full-text articles. Outcomes of intraoperative blood loss in milliliters and surgical time in minutes were considered two primary outcomes. Additionally, residual adenoid tissue, postoperative bleeding, postoperative complications such as infection, retained swab, postoperative dehydration, associated trauma, and velopharyngeal dysfunction were considered secondary outcomes for quantitative synthesis. We performed a qualitative review of uncommon outcomes such as pain scores, mean number of revision surgeries, pediatric sleep based on questionnaire results, nasal airway obstruction based on visual analog scale results, velopharyngeal insufficiency, injury of adjacent structures, and complete cicatrization.

We identified Medical Subject Heading (MeSH) keywords and text words. The most prevalent search terms found in abstracts and titles were “adenoid hypertrophy”, “adenoid hypertrophic”, “adenoidectomy”, “treatment modalities and adenoid hypertrophy”, “surgery for adenoid hypertrophy”, “conventional curettage technique”, and “surgical techniques for adenoidectomy”. Then, we merged these major concepts using combinations (AND, OR) relevant to the objective of the study. These keywords included “Adenoid hypertrophy” OR “Adenoid hypertrophic” OR “hypertrophied adenoids” OR “Adenoidectomy” AND “conventional curettage technique”. Moreover, to detect more research articles, we used truncation (*) with the same root word to make sure that we retrieved all potential variants of the search terms. We also applied search limits or filters to the language (English) and applied restrictions to the publication period, age group, and type of intervention to include eligible studies in the search.

### Data abstraction

We imported all appropriate research studies into the reference manager software (Endnote™) file, and each study was reviewed. We also screened titles for duplicates using this software. The full texts were not reviewed if the abstracts did not explicitly explore the study objective. Finally, we obtained and investigated the full-text articles of the remaining relevant research articles. Then, we abstracted and summarized the articles that met the eligibility criteria using a standardized proforma. After the process of removing duplicates, title screening, and abstract screening, we removed articles that were beyond the scope of this review as guided by the inclusion criteria. The bibliographies of the remaining studies were also verified and examined to avoid missing any useful studies. The process of searching the articles was performed independently by the reviewers, and their judgments and extracted summaries were matched to identify the differences and resolve them accordingly.

Independent reviewers completed a standardized data extraction sheet for the eligible research articles and extracted the characteristics of the studies. The reviewers compared the data extraction tables to ensure that the imperative findings of the eligible studies were included and pilot-tested the data extraction sheet before starting the process of data extraction. Prevailing research articles on the chosen topic were reviewed to describe objects of the data extraction proforma. Any discrepancies among the three reviewers were solved by an agreement among them. The abstracted data comprised the study name, publication year, sample size or population, country or study setting, average age and age range, sex, characteristics of the study participants, type of intervention, and parameters such as mean, standard deviation, median, and interquartile range.

### Quality assessment

Having included all randomized controlled trials in this network meta-analysis, a Cochrane Collaboration Risk of Bias Tool was used to evaluate the quality of all eligible studies [19]. The major domains that were assessed were randomization, allocation concealment, blinding of study participants, blinding of outcome assessors, and completeness of data regarding outcomes. Based on these domains, we were able to assess the degree of bias for the included studies, which was rated as low, high, or unclear. A final graph was generated to visualize the extent of bias in all eligible studies [19].

### Statistical analysis

Differences among the studied techniques were computed using the mean difference (MD) for continuous parameters and their respective 95% confidence intervals (CIs); the odds ratios (ORs) with their 95% CIs were computed for binary outcomes. For continuous parameters, we used the means and standard deviations (SDs) of the last follow-up period. For dichotomous parameters, percentages were used to compute ORs. If results were reported as the median and interquartile range for continuous parameters, then the means and SDs were estimated according to the available standard statistical guidelines [20]. We performed this network analysis with indirect and mixed comparisons using STATA version 16.0 (StataCorp, College Station, TX, USA) and Open Meta[Analyst] software. The absence of a closed loop in the network meta-analysis prevented the authors from using the inconsistency test. The results are presented as milliliters for blood loss outcomes and as minutes for the surgical time outcomes; their corresponding 95% CIs are also presented.

An inverse variance statistical method and random-effects model were used for this meta-analysis because of the differences among studies attributable to the sample size, outcome assessment, and/or tools used to measure outcomes. Heterogeneity was assessed using the I^2^ statistic. Forest plots were generated to compute the individual effect as well as pooled effects of the intervention for the primary outcomes (blood loss in milliliters and surgical time in minutes) and to assess statistical heterogeneity. Additionally, forest and funnel plots were generated for secondary outcomes such as residual adenoid tissue, postoperative bleeding, and various postoperative complications. Furthermore, to explore the possible ranking of the different interventions and obtain a treatment hierarchy, the surface under the cumulative ranking (SUCRA) curves and the mean ranks were estimated for two primary outcomes. When the intervention has a SUCRA value close to 1 and a low mean rank score, the results of that intervention will be better (i.e., less likely to cause a large amount of blood loss or more likely to involve shorter surgical time). *P* < 0.05 was considered statistically significant.

## Results

### Search results

**Fig. 1:**
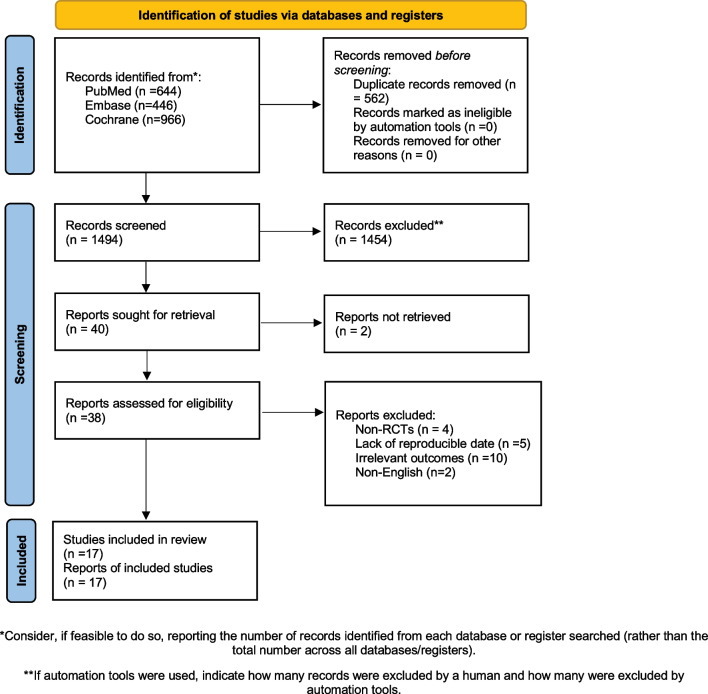
Flow diagram showing the process of screening and identifying studies for systematic review and net-work meta-analysis

The chosen articles were initially screened by titles. Then, they were screened by abstracts. Finally, the full-text articles were assessed. Our initial search identified 2523 citations in different databases; however, 2270 records were excluded because their titles and abstracts were not relevant. Of the remaining 33 unique full-text articles, 16 were further excluded because they did not meet the eligibility criteria. Finally, we included 17 articles for qualitative synthesis as a part of the systematic review; these 17 articles were also included in the quantitative synthesis as shown in the PRISMA flow diagram for screening studies (Fig. 1).

### Characteristics of the eligible studies

All studies were randomized controlled trials conducted in countries such as the United States (n = 5), India (n = 4), Turkey (n = 3), Saudi Arabia (n = 1), South Africa (n = 1), Iraq (n = 1), and Israel (n = 1) (Table 1). One study was conducted in 2020, one in 2019, one in 2018, three in 2012, one in 2010, one in 2009, one in 2007, one in 2004, one in 2000, and one in 1997 (Table 1). The overall sample size of all included research studies varied between 32 and 600, with a similar distribution among patients who were randomized to different surgical treatment modalities for adenoidectomy. The age of the study participants ranged between 1.2 months and 18 years, with varying proportions of males and females in the intervention and control groups. While reviewing the studies, we found a series of surgical techniques that were compared with conventional curettage adenoidectomy. These interventions included suction diathermy, suction cautery with antistick, endoscope-assisted coblation adenoidectomy, endoscopic-assisted microdebrider adenoidectomy, endoscopic-assisted adenoidectomy with a curette, endoscopic-assisted adenoidectomy transnasal forceps, EMRA (suction electrocautery), and gold laser adenoidectomy. The most common technique reviewed was endoscopic-assisted adenoidectomy.

**Table 1:** Characteristics of the eligible studies that were assessed for various outcomes (n = 17)

Study name	Year	Country/Study location	Total sample size at baseline	Age in years (Range)	Gender	Characteristics of patients	Follow up time
Shorook Na'ara et al. [21]	2020	Haifa, Israel	58	1.2 to 15	M:58.6% F: 41.3%	Breating and sleeping problems	1 to 12 months
Secaattin Gu ¨ls et al. [3]	2019	Yozgat, Turkey	72	5 to 11	M: 50% F: 50%	Nasal airway obstruction with chronic postnasal discharge	1 to 7 days
Saroo Singh et al. [8]	2019	New Delhi, India	60	6 to 12	M: 78.3% F: 21.6%	Adenoid hypertrophy	3 months
Juneja et al. [10]	2018	New Delhi, India	50	5 to 12	M: 70% F: 30%	Nasal or aural symptoms due to adenoid hypertrophy	1 day to 3 months
Mularczyk et al. [23]	2018	Illinois,USA	101	< 18 years	NR	Adenoid hypertrophy	3 days
Hussein and Al-Juboori [36]	2012	Hilla, Iraq	40	4 to 20	M: 40% F: 60%	Nasal airway obstruction with sleep disordered breathing, epistaxis, discharge, and otological symptoms	Post-operative period (short follow-up): Exact duration NR
Baker et al. [28]	2012	Virginia, USA	61	1 to 12	M: 60.6% F: 39.3%	Adenoid hypertrophy	Post-operative period (short follow-up): Exact duration NR
Öztürket al [29]	2012	Istanbul, Turkey	56	1.8 to 15.6	M:54.7% F: 45.3%	Nasal airway obstruction with sleep disordered breathing and otitis media and chronic rhinosuinitis	6 months
Bradoo et al. [37]	2011	Mumbai, India	32	5 ot 13	M:54.8% F: 45.2%	Mouth breathing, adenooid facies, ear problems, and saliva drooling	3 months
Songu et al. [30]	2010	Izmir, Turkey	38	8 to 12	M:52.6% F: 47.3%	Adenoid hypertrophy	3 months
Datta et al. [24]	2009	Mumbai, India	60	6 to 12	NR	Adenoid hypertrophy	1 to 7 days
Al-Mazrou et al. [27]	2009	Riyadh, Saudi Arabia	40	3 to 17	M: 50% F: 50	Snoring, obstructive sleep apnea and enlarged adenoids	3 to 24 months
Jonas et al. [3]	2007	Cape Town, South Africa	100	1.2 months to 13 years	M: 49% F: 51%	Snoring, obstructive sleep apnea, nasal airway obstruction, chronic otitis media, and enlarged adenoids	6 months
Shapiro et al. [25]	2007	California, USA	47	2 to 16	M: 59.5% F: 38.3%	Adenoid hypertrophy	14 days
Tarantino et al. [26]	2004	Genova, Italy	600	2 to 10	NR	nasal airway obstruction, nasal dyspnea, noisy breathing, otitis media, and hearing loss	Post-operative period (short follow-up): Exact duration NR
Stanislaw et al. [31]	2000	New York, USA	177	1 to 13	M: 57.6% F: 42.3%	Adenoid hypertrophy	1 to 7 days
Clemens et al. [32]	1997	USA	34	4.9 ± 2.8 and 6.9 ± 4.1	NR	Adenoid hypertrophy	1 month

### Primary outcomes: intraoperative blood loss and surgical time

**Fig. 2:**
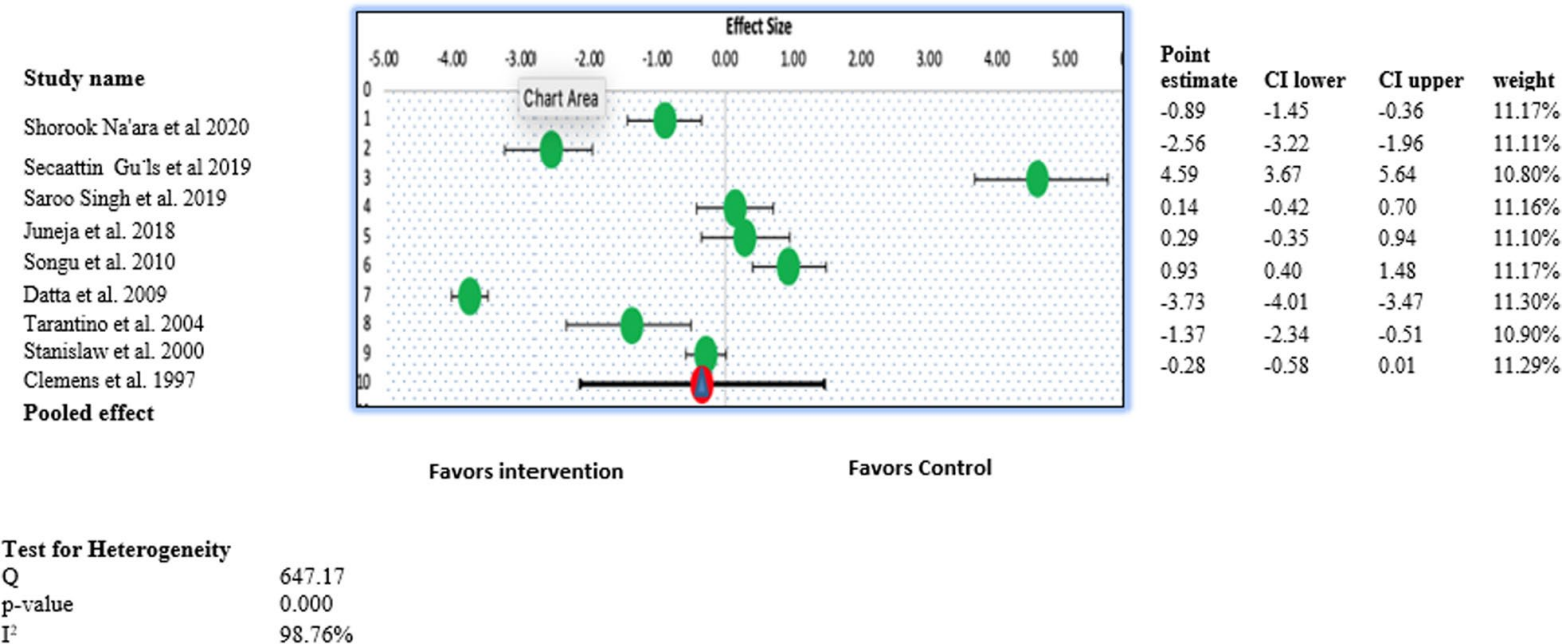
Forest plot for the individual and pooled results for Intraoperative estimated blood loss in ml during adenoidectomy

Figure 2 shows the forest plot of the pooled results of intraoperative blood loss. Overall, the forest plot demonstrated no significant difference between the intraoperative blood loss mean reduction scores of the intervention and control groups (MD, − 0.34; 95% CI − 2.01 to 1.31; *P* > 0.05) (Fig. 2). This revealed that children who underwent any procedure other than conventional curettage experienced similar blood loss, on average, compared with the control group, and the results were statistically insignificant. High heterogeneity was observed for this outcome, with an I^2^ value of 98.76% (χ^2^ = 647.17). A random-effects model was used to generate the forest plot of this outcome.

**Fig. 3:**
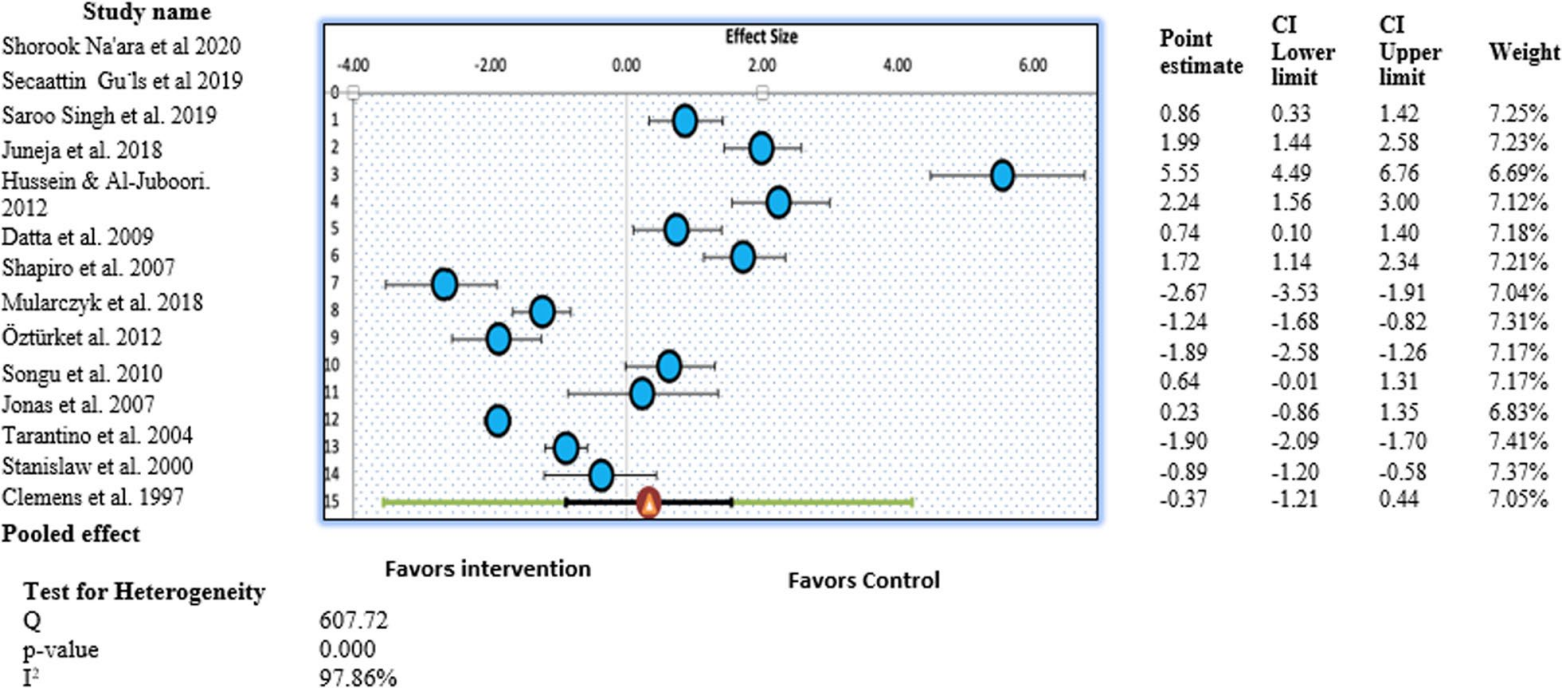
Forest plot for the individual and pooled results for the surgery time in minutes during adenoidectomy

Figure 3 illustrates the forest plot of the pooled results of the surgical time. Overall, the forest plot demonstrated no statistically significant difference in the surgical time for the intervention and control groups (MD, 0.33; 95% CI, − 1.11 to 1.92; *P* > 0.05) (Fig. 3). This revealed that study participants who underwent any procedure other than conventional curettage had similar surgical time, on an average, compared with the control group, and the results were statistically insignificant. An evaluation of the individual studies showed that curettage required less time than all other techniques, including endoscopic-assisted diathermy; however, the pooled results did not reveal any statistically significant difference. High heterogeneity was observed in this study pool, with an I^2^ value of 97.86% (χ^2^ = 607.72). A random-effects model was used to generate the forest plot and pooled results of this outcome.

### Secondary outcomes: residual tissue, postoperative complications, and postoperative bleeding

**Fig. 4:**
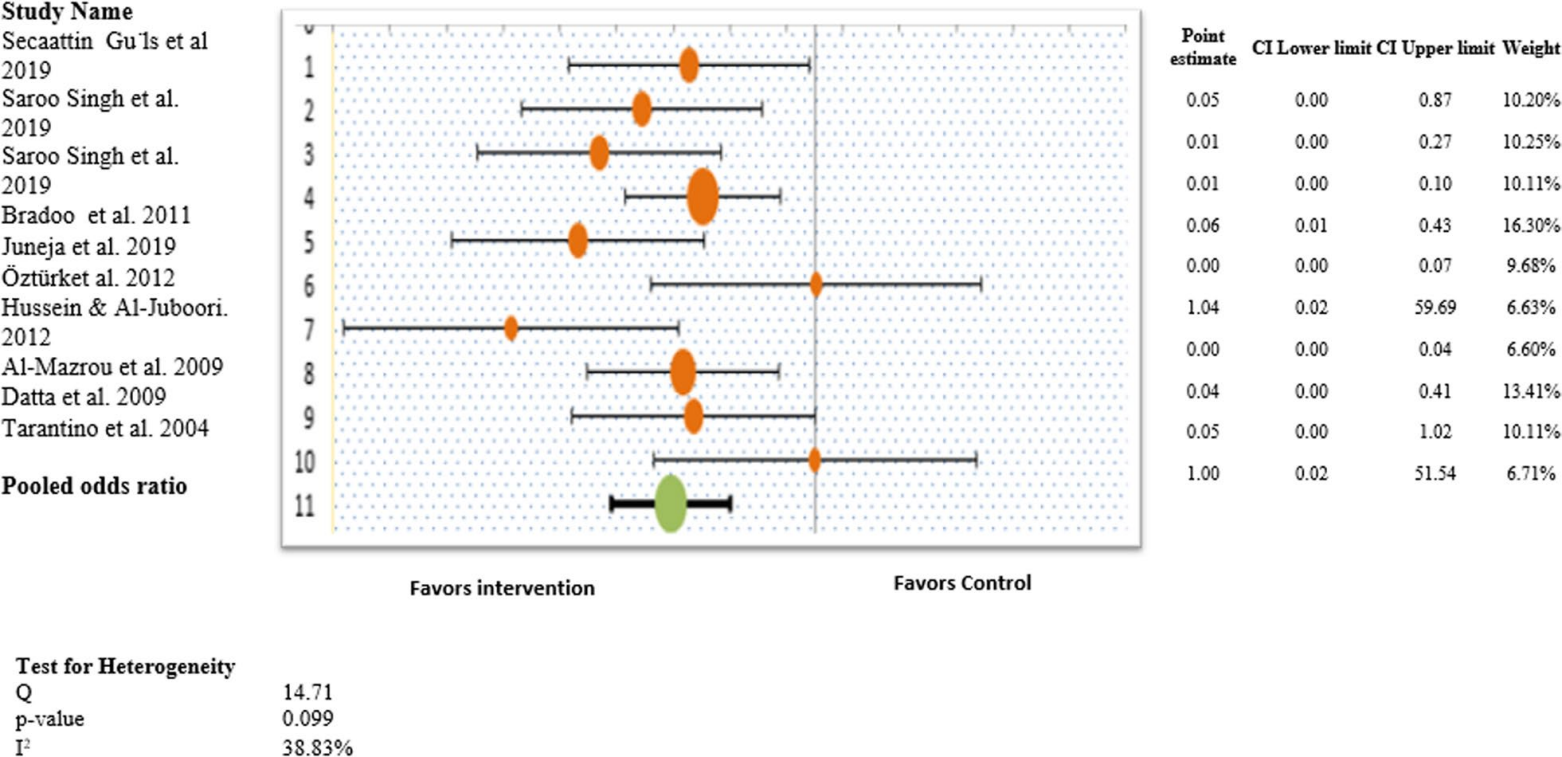
Forest plot for the individual and pooled results representing odds ratios for residual adenoid tissue percentage

Figure 4 illustrates the forest plot of the pooled results of the secondary outcome of residual adenoid tissue. Overall, the forest plot demonstrated a statistically significant difference in the ORs of residual adenoid tissue of the control and intervention groups (OR, 0.03; 95% CI, 0.01–0.15; *P* < 0.05) (Fig. 4). This revealed that study participants who underwent any procedure other than conventional curettage were at lower risk for residual adenoid tissue compared with the control group, and the results were statistically significant. More specifically, participants in the intervention group were 97% less likely to have residual adenoid tissue than children in the control group. Low heterogeneity was observed in this study pool, with an I^2^ value of 38.83% (χ^2^ = 14.71). A random-effects model was used to generate the forest plot and pooled findings of this secondary outcome.

**Fig. 5:**
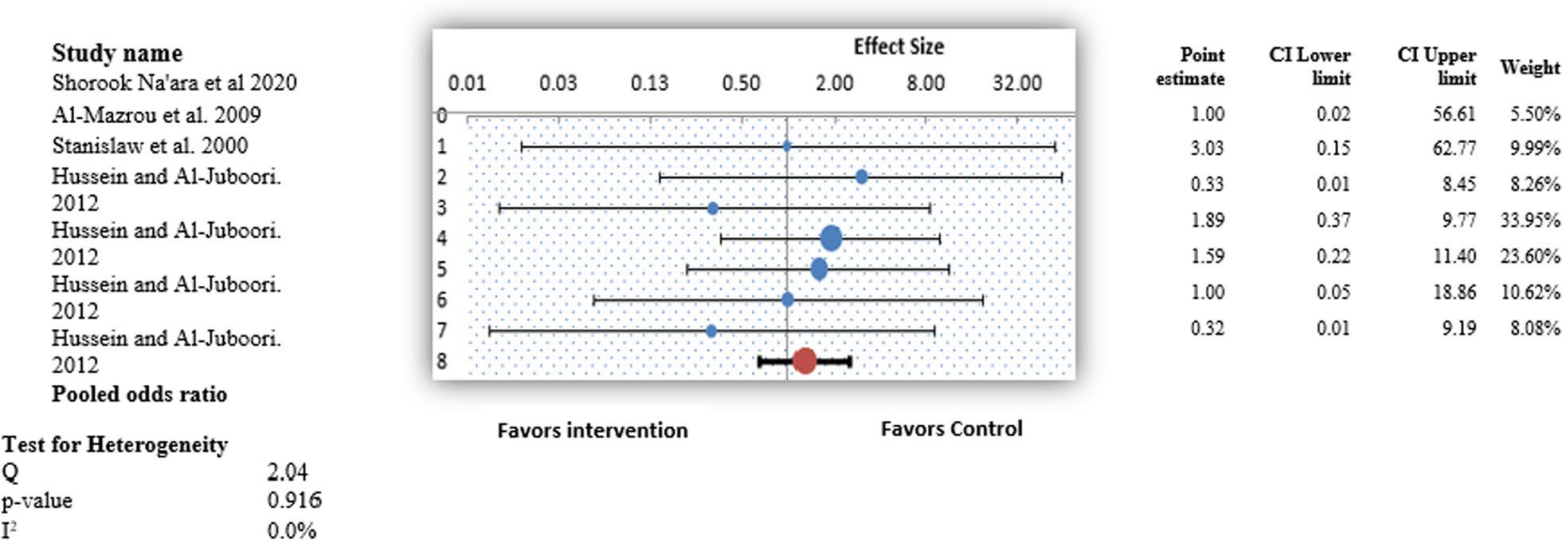
Forest plot for the individual and pooled results representing odds ratios for post operative complications

Figure 5 illustrates the forest plot of the pooled results of postoperative complications. Overall, the forest plot demonstrated no statistically significant difference among all types of postoperative complications experienced by the intervention and control groups (OR, 1.29; 95% CI 0.40–2.50; *P* > 0.05) (Fig. 5). This revealed that study participants who underwent any procedure other than conventional curettage experienced postoperative complications similar to those of the control group, and the results were statistically insignificant. No heterogeneity was observed in this study pool, with an I^2^ value of 0.0% (χ^2^ = 2.04). A random-effects model was used to generate the forest plot and pooled findings of this outcome.

**Fig. 6:**
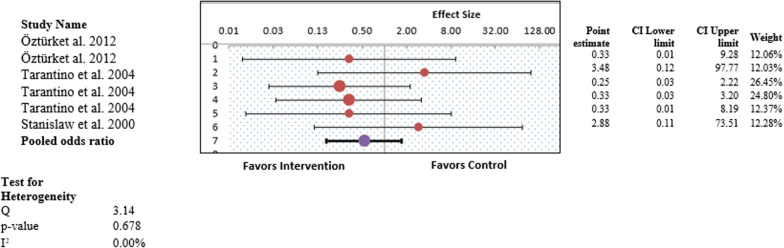
Forest plot for the individual and pooled results representing odds ratios for post operative bleeding

Figure 6 illustrates the forest plot of the pooled results of postoperative bleeding. Overall, the forest plot demonstrated no statistically significant difference among all types of postoperative complications experienced by the intervention and control groups (OR 0.53; 95% CI 0.12–1.95; *P* > 0.05) (Fig. 6). This revealed that study participants who underwent any procedure other than conventional curettage experienced postoperative bleeding similar to that of the control group, and the results were statistically insignificant. No statistically significant differences in postoperative bleeding were observed in the individual studies. No heterogeneity was observed in this study pool, with an I^2^ value of 0.0% (χ^2^ = 3.14). A random-effects model was used to generate the forest plot and pooled results for this outcome.

### Ranking of the different interventions and treatment hierarchy for primary outcomes

**Fig. 7:**
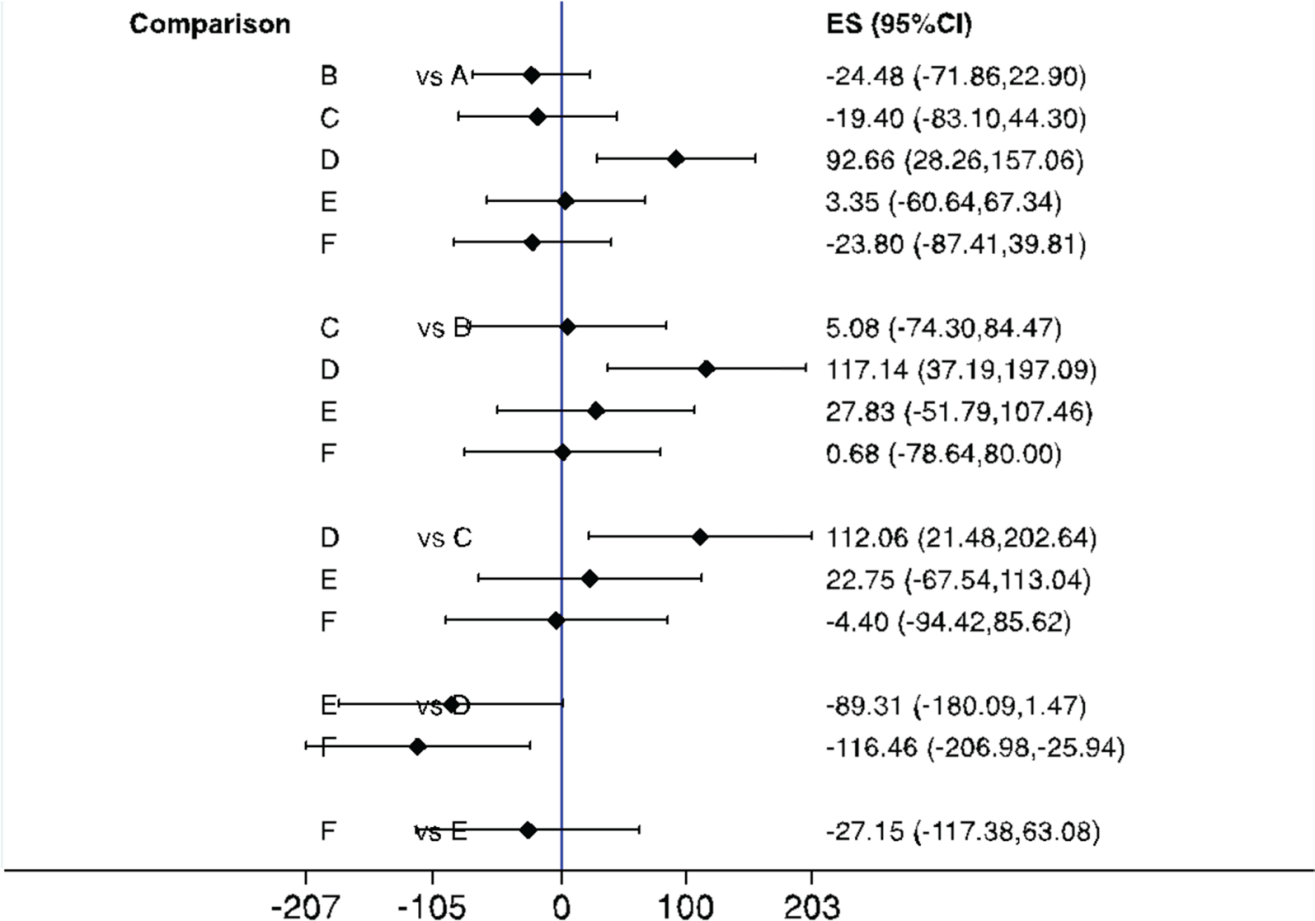
Interval plot delineating different techniques of adenoidectomy in reference to intraoperative estimated blood loss. **A** Curette, **B** Suction diathermy, **C** Endoscopic assisted coblation adenoidectomy, **D** Endoscopic assisted microdebrider adenoidectomy, **E** Endoscopic assisted adenoidectomy (transnasal forceps), **F** Electronic molecular resonance adenoidectomy EMRA (suction electrocautery). ES, Effect size; CI, Confidence interval

**Fig. 8:**
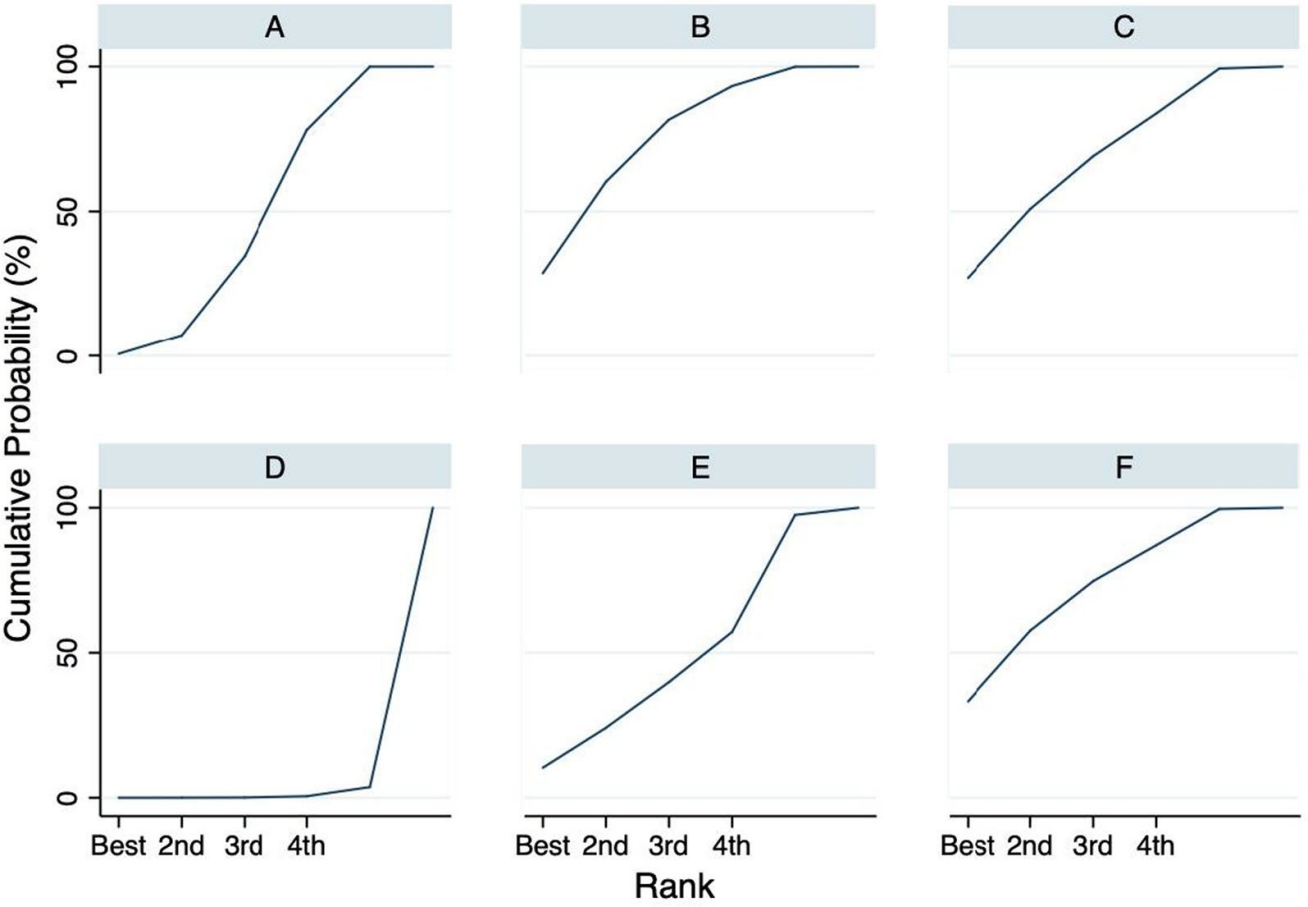
The cumulative ranking curve of intraoperative estimated blood loss outcome. The surface under the cumulative ranking curve (SUCRA) represents the ranking of treatment interventions. A higher SUCRA suggests a higher probability of being a good treatment. **A** Curette, **B** Suction diathermy, **C** Endoscopic-assisted coblation adenoidectomy, **D** Endoscopic-assisted microdebrider adenoidectomy, **E** Endoscopic-assisted adenoidectomy (transnasal forceps), **F** Electronic molecular resonance adenoidectomy EMRA (suction electrocautery)

All treatment interventions were compared to conventional curettage to evaluate intraoperative blood loss. Based on the results of a conventional meta-analysis, when compared to conventional curettage, no technique showed a statistical difference in intraoperative blood loss. However, according to the network meta-analysis, endoscopic-assisted microdebrider adenoidectomy yielded a statistically significantly greater intraoperative blood loss estimate compared with conventional curettage (MD, 92.7; 95% CI 28.3–157.1), suction diathermy (MD, 117.1; 95% CI 37.2–197.1), endoscopic-assisted coblation adenoidectomy (MD, 112.1; 95% CI 21.48–202.6), and EMRA (MD, 116.5; 95% CI 25.9–207) (Fig. 7). The ranking results showed that suction diathermy had the highest cumulative probability of being the preferred technique because it was estimated to result in the least intraoperative blood loss (Table 2 and Fig. 8).

**Table 2:** Results of network rank test of the intraoperative estimated blood loss outcome

Study and rank	Surgical technique for adenoidectomy					
1st Best	A	B	C	D	E	F
	0.6	29.6	27.2	0.0	9.5	33.1
2nd	7.3	31.0	25.3	0.0	11.3	25.1
3rd	25.4	22.5	19.3	0.2	15.7	16.9
4th	46.5	9.0	14.6	0.4	18.2	11.3
5th	20.2	7.6	13.1	3.2	42.8	13.1
Worst	0.0	0.3	0.5	96.2	2.5	0.5
Mean rank	3.8	2.3	2.6	6.0	3.8	2.5
SUCRA	0.4	0.7	0.7	0.0	0.4	0.7

**A:** Conventional Curette adenoidectomy, **B:** Suction diathermy, **C:** Endoscopic assisted coblation adenoidectomy, **D:** Endoscopic assisted microdebrider adenoidectomy, **E:** Endoscopic assisted adenoidectomy (transnasal forceps), **F:** Electronic molecular resonance adenoidectomy EMRA (suction electrocautery). SUCRA: The surface under the cumulative ranking curve

**Fig. 9:**
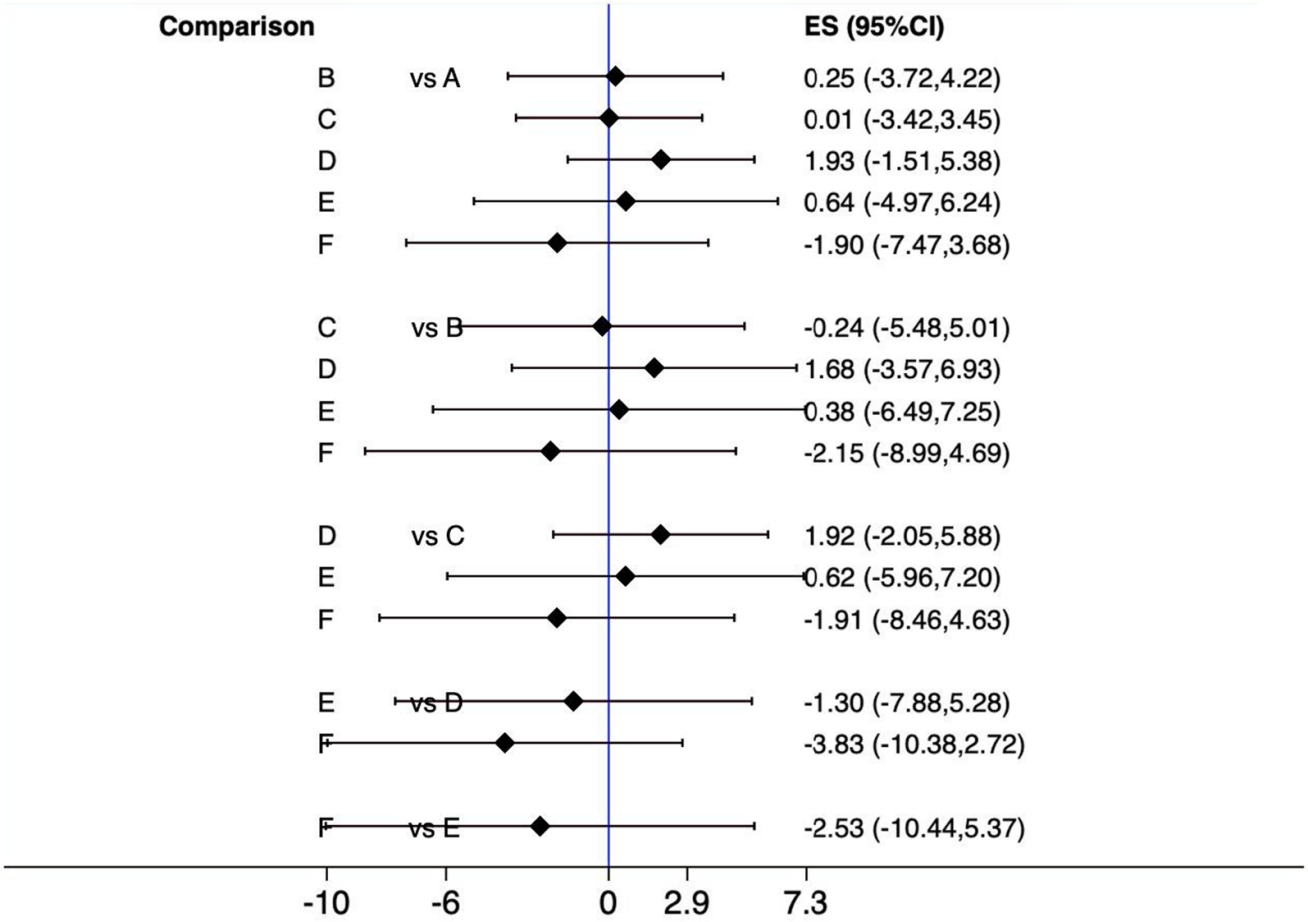
Interval plot delineating different techniques of adenoidectomy in reference to estimated procedure time. **A** Curette, **B** Suction diathermy, **C** Endoscopic assisted coblation adenoidectomy, **D** Endoscopic assisted microdebrider adenoidectomy, **E** Endoscopic assisted adenoidectomy (transnasal forceps), **F** Electronic molecular resonance adenoidectomy EMRA (suction electrocautery). ES, Effect size; CI, Confidence interval

**Fig. 10:**
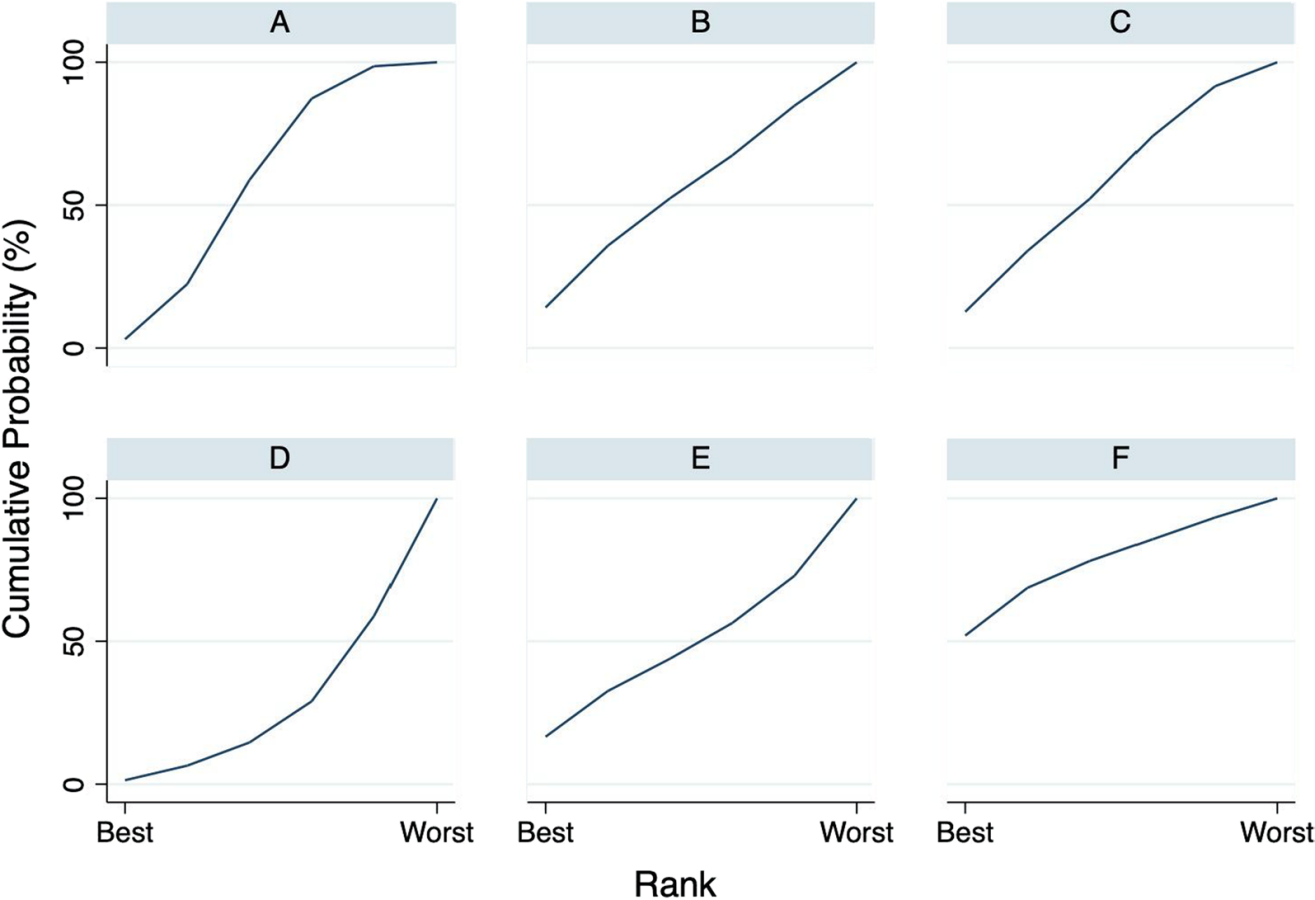
The cumulative ranking curve of estimated procedure time outcome. The surface under the cumulative ranking curve (SUCRA) represents the ranking of treatment interventions. A higher SUCRA suggests a higher probability of being a good treatment. **A** Curette, **B** Suction diathermy, **C** Endoscopic-assisted coblation adenoidectomy, **D** Endoscopic-assisted microdebrider adenoidectomy, **E** Endoscopic-assisted adenoidectomy (transnasal forceps), **F** Electronic molecular resonance adenoidectomy EMRA (suction electrocautery)

The results of the interval plot of the estimated surgical time (Fig. 9) showed no statistically significant difference among the interventions. A further analysis using SUCRA rankings indicated that EMRA was more likely to result in the shortest surgical time (mean rank, 2.2; SUCRA score, 0.8) (Table 3 and Fig. 9). However, according to the same analysis, endoscopic-assisted microdebrider adenoidectomy was likely to be the least favorable procedure (because it had the longest surgical time) among all studied techniques (mean rank, 4.9; SUCRA score, 0.2) (Fig. 10).

**Table 3:** Results of network rank test of the estimated procedure time outcome

Study and rank	Surgical technique for adenoidectomy					
1st Best	A	B	C	D	E	F
	3.3	14.2	11.7	1.6	15.4	53.8
2nd	19.7	17.7	25.7	4.2	17.3	15.4
3rd	37.8	16.2	18.6	8.6	9.8	9.0
4th	27.3	19.0	18.7	14.7	12.1	8.2
5th	10.1	18.2	19.4	27.7	18.0	6.6
Worst	1.8	14.7	5.9	43.2	27.4	7.0
Mean Rank	3.3	3.5	3.3	4.9	3.8	2.2
SUCRA	0.5	0.5	0.5	0.2	0.4	0.8

**A:** Conventional Curette adenoidectomy, **B:** Suction diathermy, **C:** Endoscopic assisted coblation adenoidectomy, **D:** Endoscopic assisted microdebrider adenoidectomy, **E:** Endoscopic assisted adenoidectomy (transnasal forceps), **F:** Electronic molecular resonance adenoidectomy EMRA (suction electrocautery). SUCRA: The surface under the cumulative ranking curve

### Qualitative synthesis performed for uncommon outcomes

A qualitative synthesis was performed for uncommon outcomes, such as pain score, mean number of handbacks, and mean recovery time (Table 4). Shorook Na’ara et al. observed changes in pediatric sleep according to the questionnaire results; these changes improved to a greater extent in the intervention group (+ 0.31 points) than in the control group (+ 0.22 points) (*P* = 0.009) [21]. However, they did not observe a difference in the adenoid size between the intervention group (3.12) and control group (3.2) (*P* = 0.6). However, Jonas et al. reported that the mean adenoid sizes were 1.5 ± 0.75 in the intervention group and 1.9 ± 0.82 in the control group at the 6-month evaluation [22]. Secaattin Guls et al. [3], Juneja et al. [10], Mularczyk et al. [23], Datta et al. [24], and Shapiro et al. [25] studied the differences in the mean pain scores; however, varied findings were reported by these different studies (Table 4). Tarantino et al. found that complete cicatrization, as indicated by the complete absence of the pseudomembrane, was achieved in 92% of patients in the intervention group and 45.3% of patients in the control group (*P* < 0.0001) [26]. Al-Mazrou et al. reported that 28.5% of patients in the curettage group experienced injury of the adjacent structures and only 11.5% of patients in the trans-nasal endoscopic powered adenoidectomy group experienced injured structures [27].

**Table 4:** Qualitative synthesis for other uncommon outcomes (n = 17)

Study name	Year	Findings for other outcomes
Shorook Na'ara et al	2020	Change in the paediatric sleep questionairre was noted as primar outcome, which improved to a greater extent in the intevention group (+ 0.31 points) than contorl group (+ 0.22 points) with a *P*-value of 0.009. Adenoid size was 3.12 in the intervention arm and 3.2 in the control arm (*P*:0.6)
Secaattin Gu ¨ls et al	2019	There was significant difference in the pain socre between two groups at first (*P*: < 0.002) and second post-operative days (*P*: < 0.003)
Saroo Singh et al	2019	The mean recovery time in the intervention group was 36 h with SD of 12.205, while it was 33.60 with SD of 11.95 in the contorl group with a *P*-value of 0.445. Postopertaive pain was higher significantly in control group than intervention arm
Juneja et al	2018	There was a higher post-opertaive pain score in the control group (6.20: 3 to 8) when compared with 4.24 (2 to 8) in the intervention group (*P* < 0.05)
Mularczyk et al	2018	Mean duration of pain was higher in the intervention (2.05 ± 1.12) than control group (1.53 ± 1.03), however, there was no statistically significant difference in the pain between two groups after controlling for adenoid size ((*P*:0.87)
Hussein and Al-Juboori	2012	No additional outcome was reported
Baker et al	2012	The mean number of handbacks per surgery was 1.9 and this average was 0.4 in the intervention group than control group (3.4) with a *P*-value of < 0.0001
Öztürket al	2012	Parents graded nasal airway obstruction on a visual analogue scale (VAS) ranging from 0 to 10. The VAS score improved among those who underwent curretage as well as power-asssisted endoscopic adenoidectomy. VAS score in the control arm improved from 8.63 to 2.22 after 6 months and it improved from 8.69 to 2.08 in the intervention arm. There was no statistically significant difference in the VAS between two groups (*P*:0.46). There was a significant reduction in the adenoid size in the intervention than control arm (*P* < 0.0001) 7.5% of the patients had laryngospasm and delayed anesthetical recovery. No recurrences were found in both groups at the follow-up
Bradoo et al	2011	No complications such as velopharyngeal insufficiency or post-opertaive bleeding were observed in either of the groups
Songu et al	2010	No significant difference was found in symptoms between two groups (*P* = 0.422). Symptoms were resolved in 61% of the petients in control group and 70% in the intervention group. Around 90% of the patients’s symptoms were resolved or improved in both groups
Datta et al	2009	The mean pain score was 2.63 (1.64–3.63) in control group whereas intervention group’s mean score was 2.13 (1.19 to 3.06) with no statistically significant difference. The mean recovery period in intervention group was 2.93 days and it was slightly prolonged (3.5 days) in the control group (*P* < 0.05)
Al-Mazrou et al	2009	28.5% of the pateints were founds to have injury of adjacent structures in the control arm versus 11.5% had injured structures in the control arm
Jonas et al	2007	Mean size of the adenoids was 1.5 ± 0.75 in the intervention group and 1.9 ± 0.82 in the control group at 6 months
Shapiro et al	2007	There was no statisitcally significnat difference in the pain scores between two groups after surgery (*P*:0.296) and no difference in the use of narcotic pain medication between two groups (*P*:0.982). Patients were returned to normal diets on similar post-opertaive days (*P*:0.982) and caregivers returned to normal routine on similar post-operative days (*P*:0.631)
Tarantino et al	2004	Complete cicatrization (complete absence of pseudomembrane) was met by 92% of the patients in intervention gropu versus 45.3% of the patients in the control group (*P* < 0.0001)
Stanislaw et al	2000	Intervnetion was 20% quicker and had 27% less blood loss than currete and provided better control for depth of resection with complete resection. Surgeons were greately satisfied with intervention than currete (*P* < 0.001). There was no difference in the recovery period or satisfaction of parents
Clemens et al	1997	No postopertaive complications were recorded in either of the groups

### Quality assessment

**Fig. 11:**
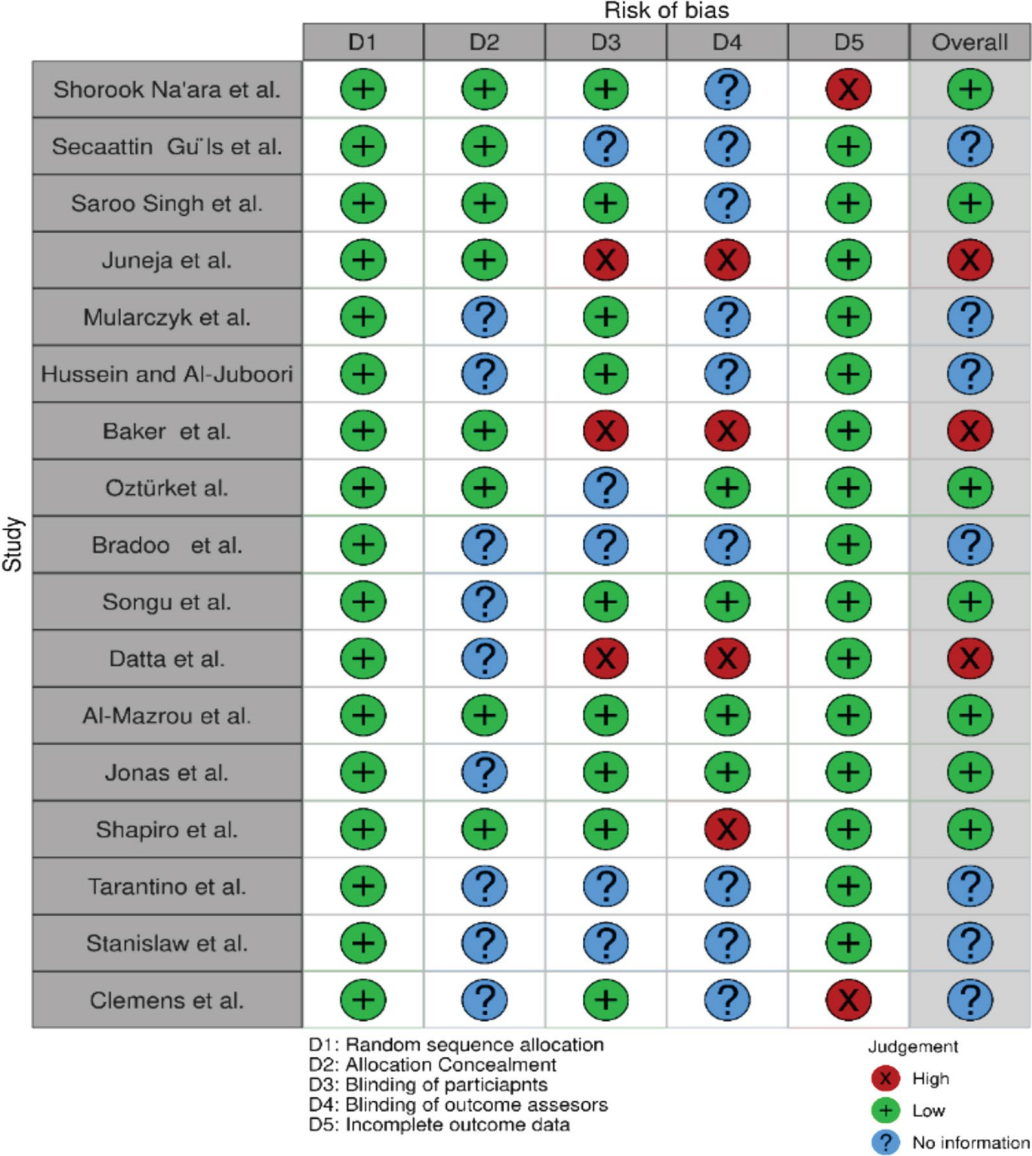
Summary for risk of bias assessment using domains for risk of bias assessment for RCTs

We assessed the study design and the quality of eligible studies. Almost all 17 studies used one-way randomization to assign study participants to the intervention group or control group. The most common technique used to randomize children was the use of a computer-generated random number table. All 17 studies mentioned randomization; therefore, they were considered to have a low risk of bias in this domain. Regarding the second domain of allocation concealment, eight of the 17 studies reported this, and none of the studies was considered to have a high risk of bias in this domain. However, for the third and fourth domains (blinding of study participants and blinding of outcome assessors), the risk of bias was higher according to studies by Juneja et al. [10], Baker et al. [28], Datta et al. [24], and Shapiro et al. [25]. Regarding the last domain (completeness of data regarding outcomes), 17 studies were rated as having a low risk of bias (Fig. 11). Overall, seven of the 17 studies were found to have a low risk of bias [3, 8, 21, 25, 27, 29, 30], three studies were found to have a high risk of bias [10, 24, 28], and the remaining studies had an unclear risk of bias because they did not provide sufficient information to rule out bias [26, 31, 32].

### Publication bias

**Fig. 12:**
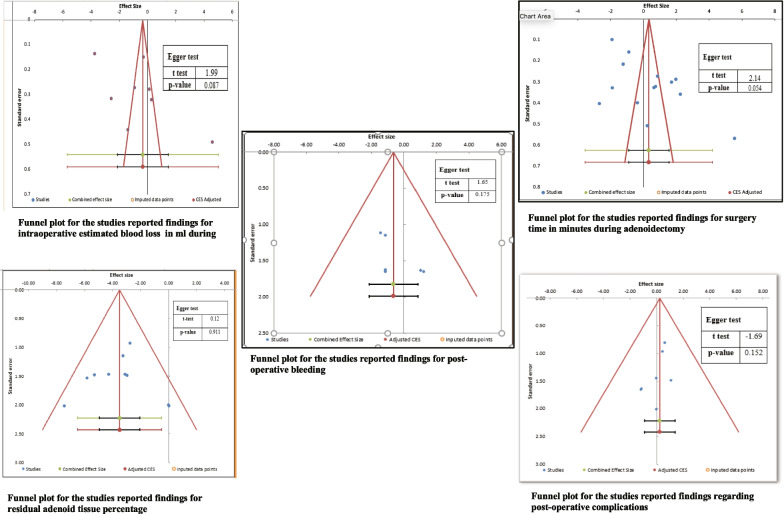
Funnel plots for the studies reported findings regarding different outcomes with eager test and *P*-values reflecting the degree of publication bias for the net-work meta-analysis

Publication bias was assessed for the different outcomes (Fig. 12). The funnel plot for intraoperative blood loss revealed no publication bias (eager test = 1.99 and *P* = 0.087). Similarly, the funnel plot for studies that reported surgical time showed no publication bias (eager test = 2.14 and *P* = 0.058). The funnel plot for studies that assessed postoperative bleeding also showed no publication bias (eager test = 1.68 and *P* = 0.175). Finally, there was no publication bias for studies that reported residual adenoid tissue (eager test = 0.12 and *P* = 0.911) and postoperative complications (eager test = − 1.69 and *P* = 0.152) (Fig. 12).

## Discussion

The growth of adenoid tissues in children may lead to varying degrees of nasal and upper airway obstructions [33]. Children with enlarged adenoids usually present with symptoms of mouth-breathing, snoring, and other complications, such as infection [11, 34]. The appropriate procedure to resolve this problem is adenoidectomy, which can improve the function of the eustachian tube and other aforementioned symptoms [35]. The most commonly performed procedure is conventional curettage adenoidectomy, followed by other techniques such as suction cautery with antistick, endoscopic-assisted coblation adenoidectomy, endoscopic-assisted microdebrider adenoidectomy, endoscopic-assisted adenoidectomy with a curettage, endoscopic-assisted adenoidectomy transnasal forceps, EMRA (suction electrocautery), and gold laser adenoidectomy [6–9]. Although all these techniques have a tendency to cause some complications, such as intraoperative blood loss, postoperative blood loss, residual adenoid tissue, pain, and others, there is a lack of evidence regarding which technique is best. Therefore, we performed a systematic review and network meta-analysis to evaluate the efficacy and safety of conventional curettage adenoidectomy compared with those of all other available adenoidectomy techniques.

This systematic review and network meta-analysis included a total of 17 studies, and the two primary outcomes were intraoperative blood loss and surgical time. The secondary outcomes were postoperative bleeding, residual adenoid tissue, and postoperative complications. Of the 17 studies, nine were included in the quantitative synthesis for intraoperative blood loss, 14 in the quantitative synthesis for surgical time, 10 in the quantitative synthesis for residual adenoid tissue, seven in the quantitative synthesis for postoperative complications, and six in the quantitative synthesis for postoperative bleeding. No statistically significant difference was observed between conventional curettage adenoidectomy and other techniques in terms of intraoperative blood loss, surgical time, postoperative complications, and postoperative bleeding. However, we found that children who underwent other techniques were 97% less likely to have residual adenoid tissue than children who underwent conventional curettage adenoidectomy. An assessment of the ranking of different interventions and treatment hierarchy for primary outcomes indicated that endoscopic-assisted microdebrider adenoidectomy had a statistically significantly greater estimate of intraoperative blood loss than conventional curettage, suction diathermy, endoscopic-assisted coblation adenoidectomy, and EMRA. Suction diathermy had the highest cumulative probability of being the preferred technique because it had the least estimated intraoperative blood loss. Furthermore, EMRA was more likely to result in the shortest surgical time, and endoscopic-assisted microdebrider adenoidectomy was likely to be the least favorable procedure (i.e., with the longest estimated surgical time) among all the studied techniques.

Our findings regarding intraoperative blood loss contradict those of a previously conducted meta-analysis that demonstrated that conventional curettage adenoidectomy leads to more intraoperative blood loss than endoscopic-assisted adenoidectomy [17]. This difference could be explained by the difference in the number of studies included in the two meta-analyses. The previously conducted meta-analysis included seven studies, and the current network meta-analysis included 17 studies. The previously conducted meta-analysis compared only two techniques (endoscopic-assisted adenoidectomy versus conventional curettage adenoidectomy) and used a conventional meta-analysis, whereas the current network meta-analysis compared different techniques with conventional curettage adenoidectomy [17]. However, findings regarding the complete removal of adenoid tissue were consistent in both meta-analyses because both found conventional curettage adenoidectomy to have lower potential to completely remove the adenoid tissue than endoscopic-assisted adenoidectomy and all other techniques assessed during the current network meta-analysis [17]. Adenoids that remain after partial removal may grow over time and lead to symptom recurrence, thereby resulting in recurrent surgical procedures with more exposure to general anesthesia. Therefore, conventional curettage adenoidectomy may not be considered a good choice for completely removing the adenoid tissue.

### Strengths and limitations

This is the first network meta-analysis of its kind and provides useful information regarding the different adenoidectomy techniques and conventional curettage adenoidectomy. We included randomized controlled trials because they are considered the gold standard in the hierarchy of study designs and provided strong evidence for comparing different adenoidectomy techniques. We provided a qualitative and quantitative review of the published randomized controlled trials and attempted to include all types of possible complications anticipated with different techniques. This meta-analysis included a relatively greater number of studies (n = 17) compared to the previously conducted meta-analysis (n = 7). Finally, the likelihood of publication bias was low, as indicated by funnel plots and eager tests for all outcomes, thereby implying that studies with both positive and negative findings have been published. However, one limitation of this meta-analysis was that all the included studies did not measure similar outcomes and were not of the same duration. Furthermore, the sample size might have been a reason for the higher heterogeneity of some outcomes observed during this meta-analysis. Because of the smaller number of studies reporting uncommon outcomes, we could not perform quantitative synthesis for pain scores, the mean number of hardbacks per surgery, pediatric sleep questionnaire results, nasal airway obstruction, velopharyngeal insufficiency, injury of adjacent structures, and complete cicatrization; however, a qualitative review was conducted for these outcomes.

## Conclusion

This systematic review and network meta-analysis found that conventional curettage, suction diathermy, endoscopic-assisted coblation adenoidectomy, and EMRA had a lower tendency for intraoperative blood loss than endoscopic-assisted microdebrider adenoidectomy. Suction diathermy can be used safely because it results in the least amount of intraoperative blood loss compared with other techniques. Finally, EMRA can be performed quickly and has the shortest surgical time. Endoscopic-assisted microdebrider adenoidectomy is likely to be the least favorable procedure among all studied techniques in terms of surgical time. As expected, conventional curettage has a relatively higher probability of residual adenoid tissue than all other techniques. However, there was no difference among conventional curettage and other techniques in terms of postoperative complications and postoperative bleeding. There is no single technique that can be considered best for all possible outcomes. Therefore, otolaryngologists need to make an appropriate choice after critically reviewing the demographic and clinical characteristics of patients. Conventional curettage may not be considered a suitable technique if surgeons need to remove all adenoid tissue. We could not perform a quantitative analysis of pain scores, recovery times, and complete cicatrization; therefore, we recommend that randomized controlled trials including the data of all possible outcomes need to be performed in the future.

## Supplementary Information

**Additional file 1.** PRISMA NMA Checklist of Items to Include When Reporting A Systematic Review Involving a Network Meta-analysis.

**Additional file 2.** PRIMSA Abstract Checklist.

**Additional file 3.** Additional Supplementary Tables.

## Data Availability

Data of this paper is available upon request.
