# Supplementary material for: Comparison of the efficacy and safety of conventional curettage adenoidectomy with those of other adenoidectomy surgical techniques: a systematic review and network meta-analysis
Source: J Otolaryngol Head Neck Surg. 2023 Mar 4;52:21. doi: 10.1186/s40463-023-00634-9 (PMC9985239; doi:10.1186/s40463-023-00634-9)
Supplement: Supplementary file 3 — Additional file 3. Additional Supplementary Tables. [file 40463_2023_634_MOESM3_ESM.docx]

**Supplementary Tables**

Interventions:

1. Suction dithermy
2. Suction cautery with antistick
3. Endoscope assisted coblation adenoidectomy
4. Endoscopic-assisted microdebrider adenoidectomy
5. Endoscopic-assisted adenoidectomy with a curette
6. Endoscopic-assisted adenoidectomy transnasal forceps
7. electronic molecular resonance adenoidectomy, EMRA (suction electrocautery)
8. Gold laser adenoidectomy

| **Table 5: Summary for findings related to the primary outcome of Intraoperative estimated blood loss in ml (n=11)** | | | | | | | | |
| --- | --- | --- | --- | --- | --- | --- | --- | --- |
| **Study ID** | **Intervention** | | | **Type of Intervention** | **Control** | | | **Type of Control** |
|  | n | Mean | SD |  | n | Mean | SD |  |
| Shorook Na'ara et al 2020 | 30 | 5.4 | 2 | Suction dithermy | 28 | 8 | 3.6 | curette |
| Secaattin Gu ̈ls et al 2019 | 36 | 24.3 | 5.1 | Endoscope assisted coblation adenoidectomy | 36 | 43.7 | 9.3 | curette |
| Saroo Singh et al. 2019 | 30 | 149.33 | 20.83 | Endoscopic assisted microdebrider adenodidectomy | 30 | 56.67 | 18.95 | Curette |
| Juneja et al. 2018* | 25 | 49.00 | 20 | Endoscopic assisted powered  adenoidectomy | 25 | 46.80 | 8 | Curette |
| Bradoo et al. 2011 | 16 | 38 | NR | Endoscopic assisted  adenoidectomy with curette | 16 | 33 | NR | Curette |
| Songu et al. 2010 | 20 | 26.32 | 13.48 | Endoscopic assisted adenoidectomy (transnasal forceps) | 18 | 22.97 | 8.37 | Curette |
| Datta et al. 2009* | 30 | 31.67 | 12.5 | Endoscopic assisted powered  adenoidectomy | 30 | 21 | 10 | Curette |
| Al-Mazrou et al. 2009 | 26 | 8.2 | NR | Endoscopic assisted microdebrider adenodidectomy | 14 | 22.1 | NR | Curette |
| Tarantino et al. 2004 | 296 | 1.2 | 0.6 | electronic molecular resonance adenoidectomy, EMRA (suction electrocautery) | 294 | 25 | 9 | Curette |
| Stanislaw et al. 2000* | 90 | 17.5 | 22 | Power- assisted adenoidectomy (PAA) | 87 | 24.0 | 23.75 | Curette |
| Clemens et al. 1997 | 12 | 3.75 | 6.4 | Suction dithermy | 12 | 54.5 | 50 | Curette |

*SD calculated using range

| **Table 6: Summary for findings related to the primary outcome of surgery time in minutes**  **(n=17)** | | | | | | | | |
| --- | --- | --- | --- | --- | --- | --- | --- | --- |
| **Study ID** | **Intervention** | | | **Type of Intervention** | **Control** | | | **Type of Control** |
|  | **n** | **Mean** | **SD** |  | **n** | **Mean** | **SD** |  |
| Shorook Na'ara et al 2020 | 30 | 9.4 | 2.2 | Suction dithermy | 28 | 6.6 | 4 | curette |
| Secaattin Gu ̈ls et al 2019 | 36 | 25.7 | 7.9 | Endoscope assisted coblation adenoidectomy | 36 | 12.1 | 5.4 | curette |
| Saroo Singh et al. 2019 | 30 | 62.67 | 6.91 | Endoscopic assisted microdebrider adenodidectomy | 30 | 26.83 | 5.79 | Curette |
| Juneja et al. 2018***** | 25 | 34.08 | 7.1 | Endoscopic assisted powered  adenoidectomy | 25 | 19.80 | 5.3 | Curette |
| Hussein and Al-Juboori. 2012***** | 20 | 42.7 | 18 | Endoscopic assisted adenoidectomy | 20 | 32.5 | 6.5 | Curette |
| Bradoo et al. 2011 | 16 | 14 | NR | Endoscopic assisted adenoidectomy with curette | 16 | 9 | NR | Curette |
| Datta et al. 2009***** | 30 | 39.3 | 7 | Endoscopic assisted powered  adenoidectomy | 30 | 29.3 | 4.1 | Curette |
| Shapiro et al. 2007 | 23 | 2.6 | 0.6 | Endoscope assisted coblation adenoidectomy | 23 | 4.1 | 0.5 | Curette |
| Mularczyk et al. 2018 | 50 | 5.50 | 2.07 | Endoscope assisted coblation adenoidectomy | 51 | 9.47 | 3.98 | microdebrider adenoidectomy with touch-up electrocautery |
| Öztürket al. 2012***** | 26 | 11.82  Median=12 | 1.0 | Endoscopic assisted microdebrider adenodidectomy | 27 | 16.52  Median= 16 | 2.75 | Curette |
| Baker et al. 2012 | 31 | 5.78 | NR | Suction cautery with antistick | 30 | 7.53 | NR | Suction cautery |
| Songu et al. 2010 | 20 | 12.02 | 8.89 | Endoscopic assisted adenoidectomy transnasal forceps | 18 | 7.15 | 5.56 | Curette |
| Al-Mazrou et al. 2009 | 26 | 6.1 | NR | Endoscopic assisted microdebrider adenodidectomy | 14 | 12.3 |  | Curette |
| Jonas et al. 2007***** | 8 | 8.6 | 4.7 | Suction diathermy | 6 | 7.5 | 4 | Curette |
| Tarantino et al. 2004 | 296 | 6 | 2 | electronic molecular resonance adenoidectomy, EMRA (suction electrocautery) | 294 | 12 | 4 | Curette |
| Stanislaw et al. 2000***** | 90 | 10.21 | 2.6 | Endoscopic assisted microdebrider adenodidectomy | 87 | 13.6 | 3.0 | Curette |
| Clemens et al. 1997 | 12 | 45.4 | 11 | Electrocautery ablation adenoidectomy (suction cautery | 12 | 50 | 13 | Curette |

*SD calculated using range

| **Table 7: Summary of findings regarding postoperative complications percentages (n=4)** | | | | | | | |
| --- | --- | --- | --- | --- | --- | --- | --- |
| **Study ID** | **Intervention** | | **Type of Intervention** | **Control** | | **Type of Control** | **Complication** |
|  | **n** | **Percentage** |  | **n** | **Percentage** |  |  |
| Shorook Na'ara et al 2020 | 30 | 0 | Suction dithermy | 28 | 0 | curette | No intraopertaive and post-opertaive complications in both groups. |
| Al-Mazrou et al. 2009 | 26 | 11.53 | Endoscopic-assisted microdebrider adenodidectomy | 14 | 4 | Curette | Injury to adjacent structures |
| Stanislaw et al. 2000 | 90 | 0 | Endoscopic-assisted microdebrider adenodidectomy | 87 | 1.1 | Curette | Postop dehydration |
| Hussein and Al-Juboori. 2012 | 20 | 25 | Endoscopic-assisted adenoidectomy | 20 | 15 | Curette | Associated trauma |
|  |  | 15 |  |  | 10 |  | Velopharyngeal dysfunction |
|  |  | 5 |  |  | 5 |  | Infection |
|  |  | 0 |  |  | 5 |  | Retained Swab |

| **Table 8: Summary of findings regarding post-operative bleeding (n=3)** | | | | | | | | |
| --- | --- | --- | --- | --- | --- | --- | --- | --- |
| **Study ID** | **Intervention** | | **Type of Intervention** | **Control** | | **Type of Control** | **Comments** |  |
|  | **n** | **percentage** |  | **n** | **percentage** |  |  |  |
| Öztürket al. 2012 | 26 | 0 | Endoscopic assisted microdebrider adenodidectomy | 27 | 3.7 | Curette | Immediate postop bleed |  |
| Öztürket al. 2012 | 26 | 3.8 | Endoscopic assisted microdebrider adenodidectomy | 27 | 0 | Curette | Delayed postop bleed |  |
| Tarantino et al. 2004 | 296 | 0.34 | electronic molecular resonance adenoidectomy, EMRA (suction electrocautery) | 294 | 1.36 | Curette | Overall bleeding |  |
| Tarantino et al. 2004 | 296 | 0.34 | electronic molecular resonance adenoidectomy, EMRA (suction electrocautery) | 294 | 1.02 | Curette | Early bleeding postop |  |
| Tarantino et al. 2004 | 296 | 0 | electronic molecular resonance adenoidectomy, EMRA (suction electrocautery) | 294 | 0.34 | Curette | Late postop bleed |  |
| Stanislaw et al. 2000 | 90 | 1.1 | Endoscopic assisted microdebrider adenodidectomy | 87 | 0 | Curette | Early postop bleeding |  |

| **Table 9:** **Summary of findings regarding** **residual adenoid tissue percentage (n=9)** | | | | | | | |
| --- | --- | --- | --- | --- | --- | --- | --- |
| **Study ID** | **Intervention** | | **Type of Intervention** | **Control** | | **Type of Control** | **Examination time** |
|  | n | Percentage |  | n | Percentage |  |  |
| Secaattin Gu ̈ls et al 2019 | 36 | 0 | Endoscope assisted coblation adenoidectomy | 36 | 22.2 | curette | Examination at 1 month postop |
| Saroo Singh et al. 2019 | 30 | 0 | Endoscopic assisted microdebrider adenodidectomy | 30 | 53.3 | curette | Postop examination |
| Saroo Singh et al. 2019 | 30 | 0 | Endoscopic assisted microdebrider adenodidectomy | 30 | 77 | curette | Endoscopy at 3 months postop |
| Bradoo et al. 2011 | 16 | 31.2 | Endoscopic assisted adenoidectomy with curette | 16 | 87.5 | Curette | 3 months postop |
| Juneja et al. 2019 | 25 | 0 | Endoscopic assisted microdebrider adenodidectomy | 25 | 88 | Curette | 1 day to 3 months |
| Öztürket al. 2012 | 26 | 0 | Endoscopic assisted microdebrider adenodidectomy | 27 | 0 | Curette | 6 months postop |
| Hussein and Al-Juboori. 2012 | 20 | 0 | Endoscopic assisted adenoidectomy | 20 | 20 | Curette | Post-operative period (short follow-up): Exact duration NR |
| Al-Mazrou et al. 2009 | 26 | 3.8 | Endoscopic assisted microdebrider adenodidectomy | 14 | 50 | Curette | Residual postop tissue |
| Datta et al.2009 | 30 | 0 | Endoscopic assisted powered  adenoidectomy | 30 | 23 |  | 1 to 7 days |
| Tarantino et al. 2004 | 150 | 0 | electronic molecular resonance adenoidectomy, EMRA (suction electrocautery) | 150 | 0 | Curette | 10 days postop by endoscopy |
